# A hypothesis for robust polarization vision: an example from the Australian imperial blue butterfly, *Jalmenus evagoras*

**DOI:** 10.1242/jeb.244515

**Published:** 2023-04-13

**Authors:** Richard A. Rabideau Childers, Gary D. Bernard, Heqing Huang, Cheng-Chia Tsai, Mary Caswell Stoddard, Benedict G. Hogan, Joel S. F. Greenwood, Edward R. Soucy, Mark Cornwall, Matthew Lek Min Lim, Marjorie A. Liénard, Nanfang Yu, Naomi E. Pierce

**Affiliations:** ^1^ Department of Organismic & Evolutionary Biology, Harvard University, Cambridge, MA 02138, USA; ^2^ Museum of Comparative Zoology, Harvard University, Cambridge, MA 02138, USA; ^3^ Department of Electrical & Computer Engineering, University of Washington, Seattle, WA 98195, USA; ^4^ Department of Applied Physics & Applied Mathematics, Columbia University, New York, NY 10027, USA; ^5^ Department of Ecology & Evolutionary Biology, Princeton University, Princeton, NJ 08544, USA; ^6^ Center for Brain Science, Harvard University, 52 Oxford St - room 331, Cambridge, MA 02138, USA; ^7^ Neurotechnology Core, Kavli Institute for Neuroscience, Yale University School of Medicine, New Haven, CT 06510, USA; ^8^ Department of Biological Sciences, National University of Singapore, 16 Science Drive 4, Singapore 117558; ^9^ The Broad Institute of MIT and Harvard, Cambridge, MA 02142, USA; ^10^ Department of Biology, Lund University, 22362 Lund, Sweden

**Keywords:** Butterfly vision, Eyeshine, Edge detection, Polarization detection, Lycaenidae, Polarization vision

## Abstract

The Australian lycaenid butterfly *Jalmenus evagoras* has iridescent wings that are sexually dimorphic, spectrally and in their degree of polarization, suggesting that these properties are likely to be important in mate recognition. We first describe the results of a field experiment showing that free-flying individuals of *J. evagoras* discriminate between visual stimuli that vary in polarization content in blue wavelengths but not in others. We then present detailed reflectance spectrophotometry measurements of the polarization content of male and female wings, showing that female wings exhibit blue-shifted reflectance, with a lower degree of polarization relative to male wings. Finally, we describe a novel method for measuring alignment of ommatidial arrays: by measuring variation of depolarized eyeshine intensity from patches of ommatidia as a function of eye rotation, we show that (a) individual rhabdoms contain mutually perpendicular microvilli; (b) many rhabdoms in the array have their microvilli misaligned with respect to neighboring rhabdoms by as much as 45 deg; and (c) the misaligned ommatidia are useful for robust polarization detection. By mapping the distribution of the ommatidial misalignments in eye patches of *J. evagoras*, we show that males and females exhibit differences in the extent to which ommatidia are aligned. Both the number of misaligned ommatidia suitable for robust polarization detection and the number of aligned ommatidia suitable for edge detection vary with respect to both sex and eye patch elevation. Thus, *J. evagoras* exhibits finely tuned ommatidial arrays suitable for perception of polarized signals, likely to match sex-specific life history differences in the utility of polarized signals.

## Teaser

**Highlighted Article:** A novel model for robust polarization vision in butterflies based on the alignment of ommatidial arrays, supported by behavioral, spectroscopic and histological evidence from the Australian butterfly *Jalmenus evagoras*.

## INTRODUCTION

Butterflies detect and respond to patterns on each other's wings in the context of social interactions, particularly sexual selection, which can involve both interspecific competition and intraspecific mate signaling. Behavioral experiments by Fordyce et al. (2002), building on the work of Pellmyr (1983), showed that female wing pattern differences in two recently diverged lycaenid species, *Lycaeides idas* and *Lycaeides melissa*, act as effective mate recognition signals that contribute to associative mating. Their experimental assay measured the behavior of patrolling males initiating courtship with choices selected from arrays of female decoys made from pinned specimens mixed with paper models on which wing pattern elements were manipulated. The first demonstration that polarized light was used as a butterfly mating signal was nearly two decades ago in *Heliconius cydno*, where experiments showed that flying males approaching female wings were attracted to the polarized light reflected from female iridescent wing scales (Sweeney et al., 2003).

The utility of polarized light as a signal extends beyond the context of mating (Marshall et al., 2019). There is an extensive literature on the dorsal rim areas of insect eyes, which are specialized for viewing polarized skylight (Labhart and Meyer, 1999). Polarized ultraviolet (UV, <400 nm) light has been well studied in the context of navigation in monarch butterflies (Heinze and Reppert, 2011; Labhart et al., 2009). Kelber (1999) showed that ovipositing female *Papilio aegeus* use a combination of color and polarized reflection to choose oviposition sites. Later, Kelber et al. (2001) found that *Papilio xuthus* can discriminate easily between two polarized light cues that differ only in polarization angle, in the context of both oviposition and feeding behaviors. In an extensive series of choice experiments with trained, freely flying *Papilio* spp., involving both color and polarization, they found that perceived colors are influenced by changes in polarization content of the stimuli. They concluded that polarization and color are processed in the same visual pathway, and that polarization can induce a change in perceived color, with an additional, smaller influence of intensity. Polarized reflected light from the wings of 144 species from four nymphalid subfamilies was examined by Douglas et al. (2007) with reference to the light environments where they naturally occur; most of the species that did not exhibit polarized reflectance patterns inhabited open light environments, suggesting that closed or complex light environments play a role in the utility of displaying and presumably perceiving polarized light.

In a study of object-based polarization vision in decapods, How and Marshall (2014) considered detection and discrimination of a polarized object against a differently polarized background. They present a computational model composed of two-channel, orthogonal photoreceptor sets with rhabdomeric microvilli aligned vertically and horizontally with the outside world. They conclude that horizontal/vertical arrays are optimally designed for detecting differences in the degree, and not the angle, of polarized light under natural conditions. However, other animal species such as stomatopods, known to use multiple two-channel systems and polarized body patterns for communication, may potentially rely on three or more channel polarization vision systems (How and Marshall, 2014; Daly et al., 2016).

A review by Marshall et al. (2019) presents several additional examples of how polarized light is utilized by different invertebrate species in ecological contexts ranging from mating to food choice. Polarization in these contexts may be perceived as contrasting colors, intensities, or both, with a correspondingly diverse range of visual system complexity. In the case of *P. xuthus*, Stewart et al. (2019) conclude that these butterflies likely have a one-channel ‘monopolatic’ polarization sensitivity, finding that they cannot unambiguously distinguish the degree and angle of polarization, and that they conflate brightness and polarization cues.

In this paper, we first demonstrate that individuals of *Jalmenus evagoras* differentiate polarization cues in some wavelengths but not others, by employing preference tests in the field of free-flying adults given a choice to inspect paired robots with actively beating wings of defined optical characteristics (color and degree of polarization). We also assessed the degree of polarization of male and female wings, finding that they differ significantly in their polarization content.

Next, we investigated how *Jalmenus* ommatidial arrays are designed for polarization detection by measuring depolarized eyeshine from patches of ommatidia as a function of eye rotation θ. We conclude that the birefringent rhabdoms creating depolarization are composed of mutually perpendicular microvilli in straight rhabdoms. Minima of depolarized intensity versus θ are measures of microvillar alignment Σ. Surprisingly, we found that many rhabdoms in a measured patch are substantially misaligned. We characterized misalignment by computing the distribution function of ΔΣ, the difference in alignment of adjacent ommatidia. Processes of edge detection and motion detection require aligned ommatidia and should involve no misaligned ommatidia. Thus, we considered how the misaligned ommatidia might be useful for vision.

Each ommatidium contains photoreceptor cells having orthogonal microvilli capable of two-channel polarization detection that could drive a first-order opponent interneuron, as observed in crustaceans (How and Marshall, 2014; Waterman, 1981). This opponent interneuron design has the advantage of polarization sensitivity that is independent of intensity, a spatial receptive field that is the same as the ommatidial visual field, and good polarization sensitivity performance for angles of polarization (ɸ) that are parallel to either microvillar direction. It has the disadvantage of being polarization blind for ɸ=45 deg with respect to microvillar directions (Bernard and Wehner, 1977).

Lycaenid behavior is very dynamic, with the eyes in constant motion, rarely stationary unless perched. If polarization detection is an important modality to them, having first-order opponent interneurons that are polarization blind at an angle of polarization (AoP) of 45 deg poses a serious challenge. However, if a second-order neuron simply sums first-order outputs from a pair of adjacent ommatidia (one aligned, one misaligned), its response versus the AoP would be robust and never polarization blind. Using *J. evagoras* as a study system, we pursued this hypothesis, and present a theoretical model of robust polarization vision driven by subarrays of these pairwise, summing second-order interneurons. If enough pairs of adjacent ommatidia with substantially different microvillar alignment each connect to their second-order interneurons in this way, the information contained in the array of second-order axons would support vision of polarized patterns, responsive to any angle of polarization. Indeed, a recent study on the connectome of the lamina of a swallowtail butterfly, *P. xuthus*, found many second-order inter-ommatidial connections (Matsushita et al., 2022), which could be involved in integrating outputs from adjacent ommatidia.

We found that males and females exhibit differences in the extent to which ommatidial arrays are aligned, and observed distinct sub-arrays of ommatidia within the eye that we hypothesize are specialized for either edge detection (ED) or detection of polarized light (polarization detection, PD), based on the sub-array alignment of their ommatidial Σ. We analyzed the arrays for such specializations and show that the PD:ED ratio varies with respect to eye-patch elevation, as well as with sex.

**List of symbols and abbreviations**

AoP: angle of polarization
DoP: degree of polarization of partially polarized light: abs[(*I*_H_−*I*_V_)/(*I*_H_+*I*_V_)]
*I*_H_: intensity of horizontal polarized component of reflected light (perpendicular to the plane of incident light)
*I*_V_: intensity of vertical polarized component of reflected light (parallel to the plane of incident light)
θ: incident angle of light in wing reflectance measurement
ɸ: angle of polarization of linearly polarized light
ϕ: angle relative to the detector in wing reflectance measurement
Σ: ommatidial principal axis, aligned with rhabdomeric microvilli or perpendicular to them
ΔΣ: difference in principal axes of two adjacent ommatidia

## MATERIALS AND METHODS

### Study organism

The Australian imperial blue butterfly, *Jalmenus evagoras* (Donovan 1805), is a model system in behavioral ecology, in part because of the complex symbiosis between its juveniles and ants (Pierce et al., 1987; Pierce and Nash, 1999; Eastwood et al., 2006). This species is noted for its striking aggregation behavior of all life stages, which is thought to result from the obligate dependency on attendant ants for protection against parasites and predators. It is also noted for its unique mating behavior, which frequently involves intense competition between males (that typically outnumber females in the field) for access to females, with males patrolling across multiple trees, inspecting females and pupae that are about to emerge (Hughes et al., 2000; Elgar and Pierce, 1988). Females of *J. evagoras* are slightly larger on average than males (Elgar and Pierce, 1988).

### Polarization discrimination behavioral experiment

#### Experimental setup and site locations

Simplified mock ‘wings’ that varied in their color and polarization content (rather than attempting to be accurate representations of *Jalmenus* wings) were constructed to test the color specificity of behavioral responses to polarized light (see below). These were affixed to the ends of two counter-rotating motor-driven rods that beat the wings at a constant rate that was inspired by the common ‘rejection response’ of a female *J. evagoras* butterfly (Movie 1). These wing-beating devices were operated in pairs, with each pair controlled by a single control box that maintained their beating at the same rate. Within each trial, two sets of wings that varied in polarization content were compared against each other by hanging the robots onto trees that were being actively visited by adults of *J. evagoras* searching for ants and conspecifics, and comparing the number of times that each robot in a pair was approached by patrolling adults. Robots were positioned in full sunlight at the same height approximately 0.5 m apart on trees (and close to the largest concentrations of pupal clusters), such that any nearby adults could easily observe both robots simultaneously from above while still allowing for approaches to either one to be clearly distinguishable. In the absence of bright sunlight, few butterflies were observed to fly at all, so the vast majority of interactions occurred in full sunlight. Trials consisted of 20 min of observation, and each was run when the butterflies were active, from about 10:00 h until about 16:00 h. Thus, trials were run in a full range of angles relative to the sun: from ∼30 to 150 deg elevation following a semicircular trajectory. All trials were randomized with respect to side and run ‘blind’ so that the observer scoring approaches during a trial did not know which of the paired robots had disrupted polarization content.

**Fig. 1.**
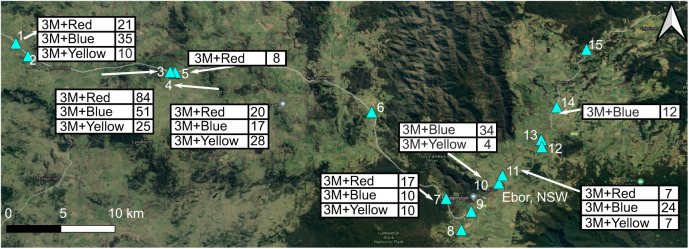
**Polarization behavioral trial sampling sites around Ebor, NSW, Australia.** Labels show wing treatments (3M+Red/Blue/Yellow) and ID numbers of field sites (marked with blue triangles) used in the polarization discrimination behavioral experiment, near Ebor, in the native range of *Jalmenus evagoras*. Google hybrid satellite base map (© Google 2022); prepared using QGIS (3.10.12).

Field experiments took place at sites near Ebor, NSW, Australia, on 16–27 February 2016, during the hours of peak butterfly activity between 10:00 h and 16:00 h. The distribution of *J. evagoras* is unusually ‘patchy’ compared with that of other butterflies, in part because the overlapping need for acacia host plants as well as specific symbiotic ants that tend and protect the juveniles creates what have been called ‘ecological islands’ of suitable habitat. At any given site, numerous butterflies and their attendant ants can be found aggregated around a cluster of host trees, but between sites, butterflies are rarely observed. Among the 15 sites initially identified to contain colonies of *J. evagoras* (Fig. 1), 10 were eventually used for experiments, with each treatment tested at between six and seven different sites. The 10 sites were in mixed savanna woodland in an area of approximately 200 km^2^ near Ebor, where *J. evagoras* is relatively common (Fig. 1). Sites were chosen by looking for patches of acacia trees (*Acacia melanoxylon*, *A. irrorata* and rarely *A. dealbata*) containing juvenile stages of *J. evagoras* tended by workers of the ant *Iridomymex mayri*, as well as numerous butterfly adults of both sexes searching for either mates or appropriate sites for oviposition. For the purposes of this experiment, we qualitatively defined an ‘approach’ response as an easily distinguishable deviation in the previous flight path of a passing individual towards a robot, coming within several centimeters of it, and often hovering for a few seconds in front of it before flying away (demonstrated in Movie 2). Most commonly, these deviations were observed to occur 0.5–1.5 m away in individuals flying above the robot from around the sides or tops of the focal trees. Thus, an individual that merely passed close to a robot but did not deviate or pause momentarily in its flight to inspect it was not considered to have made an approach. Most of these interactions occurred under bright sunshine, given that that these butterflies rapidly settle under foliage in response to shade from passing clouds, and are largely inactive on cloudy days.

#### Mock wing construction and robot design

**Fig. 2.**
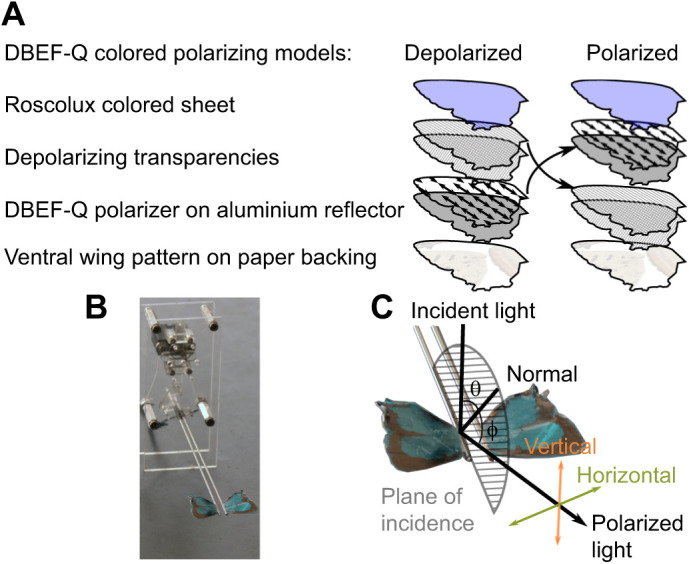
**Mock wing construction and robot assembly.** (A) Mock wing layers for polarized and depolarized DBEF-Q treatments (see Materials and Methods for further details). (B) Robotic wing beater assembly with blue (R374) DBEF-Q wing treatment affixed. (C) Diagram describing the definitions of polarization employed to fabricate and characterize model and specimen wings. ‘Horizontal’ represents polarization perpendicular to the plane of incident light (semicircle); ‘Vertical’ represents polarization parallel to the plane of incident light. Angles of incident light (θ) and detection angle (ϕ) are both relative to normal (vertical from the specimen).

The mock wings used in the above experiment consisted of a series of stacked, laser-cut layers (Fig. 2). The ventral (base) layer of both polarized and de-polarized wings was a layer of paper printed with a scan of the ventral wing surface of a *J. evagoras* female (scaled to the original wing size). De-polarized wings then had a layer of aluminium foil (which we used as a spectrally neutral surface with maximal reflectance) upon which the adhesive polarizing filter (DBEF-Q 0 deg, 3M, St Paul, MN, USA; observed in initial tests to be not UV transmissive; for additional performance details, see Boyd, 2012) had been affixed, topped with two layers of clear 92296 T LaserJet Monochrome Transparencies (HP Inc., Palo Alto, CA, USA), observed to be highly birefringent and crossed at 90 deg with respect to each other to disrupt the linear polarization of the layer below. To maintain similar physical properties between polarized and de-polarized wings, these layers were also included in polarized wings, above the base paper layer but below the aluminium foil and polarizing filter layer. Both polarized and depolarized wings had identical Roscolux (Stamford, CT, USA) transparencies of various colors (#R374: Sea Green; #R312: Canary; #G280: Fire Red) to the top layers.

In both cases, these stacks of layers were bound together and affixed to the ventral wing printouts along the edge with small strips of clear tape that were tested first to make sure that they did not disrupt polarized light (determined by rotating the tape between two orthogonal linear polarizers; little or no change in perceived brightness meant there was minimal depolarization of linearly polarized light induced by the tape). This also ensured that the polarization content of the wings they bound was not disrupted.

The reflective 3M polarizers transmitted much more light than traditional linear polarizers, allowing for much brighter color treatments, but preliminary observations showed they were opaque to UV light. The linear polarization (or disrupted polarization) of each of these wings was individually assessed by placing them on a table under a bright, noon sunlight and observing them through a rotating linear polarizer from all angles above and around the wings. Polarized wings showed strong extinction as the polarizer was rotated; a change of the detection angle with respect to the wing was indicative of strong linearly polarized reflection, whereas the polarization-disrupted wings showed no discernible extinctions.

A custom mechanical device was built to move interchangeable artificial butterfly wings with naturalistic motion. A micro-gearmotor (6–12 V DC, 30:1; ROB-12316, Sparkfun, Boulder, CO, USA) was attached to a scotch yoke style mechanical linkage to convert rotary motion into a sinusoidal wing beating motion (schematics available upon request). Each motor revolution resulted in the wings cycling through 76 deg, moving from near horizontal to near vertical back to near horizontal with mirror symmetry (Movie 2). A tethered remote control (7 m) supplied power and allowed for field adjustment of the wing beat speed (0–15 Hz) by regulating the voltage from a standard 9 V battery using a 500 Ω linear potentiometer (450D501-6-ND, Digikey, Thief River Falls, MN, USA). The speed of each unit was yoked by sending the same control voltage from the remote.

### Reflectance spectrophotometry and polarization imaging

Reflectance spectra were measured from areas ∼5 mm in diameter on the mock wings and at the highly reflective regions on the forewings and hindwings of *N*=12 female and *N*=13 male pinned pristine specimens of *J. evagoras* (specimen metadata, available from Dryad: https://doi.org/10.5061/dryad.kprr4xh6t). During these measurements, each sample was illuminated with a collimated full-spectrum (350–700 nm) beam generated from a Xenon lamp (HPLS-30-03, Thorlabs, Newton, NJ, USA), and the reflected light was collected by an integrating sphere (IS200-4, Thorlabs) and analyzed with a spectrometer (STS-VIS, OceanOptics, Largo, FL, USA). Each raw spectrum was normalized to that measured from a reflectance standard, which has reflectivity close to unity in the measurement wavelength range.

We also conducted polarization imaging experiments to study the polarization properties of the wing specimens as well as the mock wings. In these experiments, a wing sample was illuminated by a beam of collimated light produced by a blue or UV light-emitting diode (LED) at various incident angles θ, ranging from −60 to 70 deg, and photos of the sample were taken at various detection angles, ϕ, ranging from 10 to 70 deg, while keeping |θ|<ϕ, with a CMOS camera (MU300, Amscope, Irvine, CA, USA) integrated with a polarizing analyzer and a wavelength bandpass filter corresponding to the LED emission color. The sum of the incident and detection angles θ+ϕ is defined as the angular contrast. The UV LED [SST-10-UV-A130, Luminus Devices, Woburn MA, USA; emission peak wavelength of 360 nm, full-width half-maximum (FWHM) of 10 nm] was used together with a broadband UV filter (FGUV11-UV, Thorlabs; λ=245–400 nm), and the blue LED (SST-10-B-B130, Luminus Devices; emission peak λ=450 nm, FWHM of 20 nm) together with a narrowband blue filter (FB450-10, Thorlabs; center λ=450 nm, FWHM of 10 nm).

Photos were taken under UV wavelengths (360 nm) and blue wavelengths (450 nm) under various incidence and detection angles for one male and one female *J. evagoras* (shown in Fig. S1). The two wavelengths match the peak spectral sensitivity of the UV and blue photoreceptors, which we found to exhibit high polarization sensitivity in the acute zone of the eye of this butterfly species (Fig. S2).

The photos were then used to estimate degree of polarization (DoP). This was done by comparing the relative brightness (or e intensity) of images of both butterfly and mock wings (excluding background colors other than wings) taken under two orthogonal polarization conditions, *I*_H_ and *I*_V_, corresponding to the intensity of the horizontal and vertical component of reflected light, respectively (see Fig. 2C), and then both of these images were used to calculate the DoP at each combination of incident and detection angles. The DoP is defined as abs[(*I*_H_−*I*_V_)/(*I*_H_+*I*_V_)], where *I*_H_ and *I*_V_ are calculated by taking the total intensity of a polarization image, at a range of angles of incident light θ and detection angles ϕ.

### A novel micro-spectrophotometric technique to determine ommatidial alignment

To learn how microvilli are organized within rhabdoms of individual ommatidia, we developed an experimental method to determine microvillar axes of rhabdoms in ommatidia of butterflies that exhibit eyeshine, measuring the variation in depolarized ommatidial eyeshine intensity as a function of eye rotation (roll angle θ) of completely intact, living butterflies with a microscopic cone of linearly polarized incident white light.

**Fig. 3.**
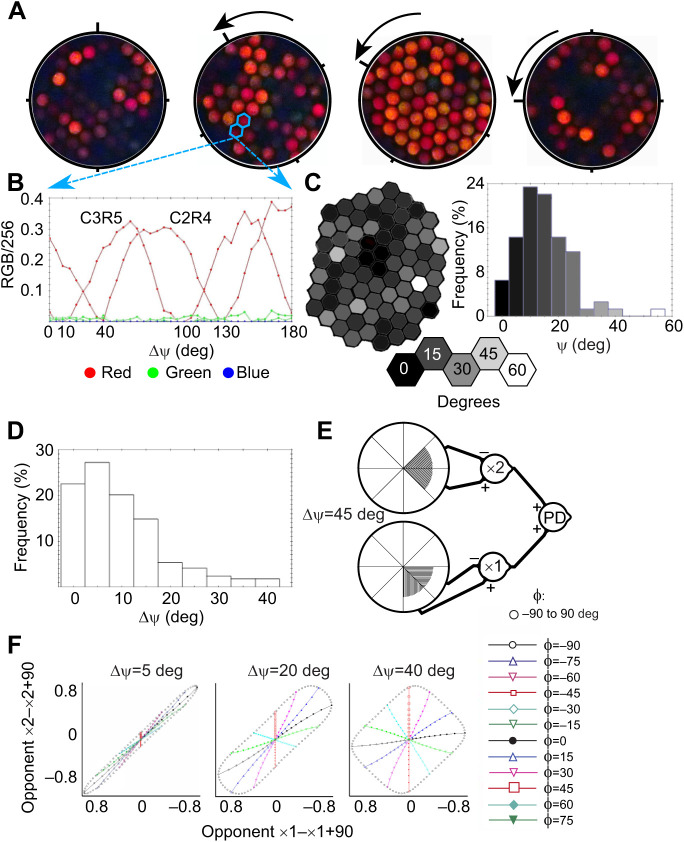
**A novel micro-spectrophotometric technique to determine ommatidial alignment.** (A) Depolarized eyeshine of an ommatidial patch (individual JEK1 male, elevation 0 deg, azimuth 10 deg) as created by a conical beam of polarized light that illuminates the rhabdom of each ommatidium, travels to its basal end, reflects from the tracheolar tapetum at its base and travels up the rhabdom and out of the eye. Microvillar birefringence in the rhabdom creates depolarized light that passes through the analyzer, a polarized filter oriented orthogonal to the polarized illumination. As the eye is rotated by angle θ beneath the incident light, the intensity of depolarized eyeshine waxes and wanes periodically in each ommatidium. (B) Depolarized eyeshine intensity of red, green and blue channels as a function of θ for two neighboring ommatidia, C2R4 and C3R5. The principal axis (Σ) of an ommatidium is here defined as the angle between 0 and 90 deg at which the depolarized eyeshine is minimal. (C) A hexagonal array showing the ommatidia and a histogram of Σ values, both gray-scaled by ommatidial Σ. (D) Distribution of ΔΣ, the difference in Σ between adjacent ommatidia. Only 22% are exactly aligned. (E) Polarization state space created by first-order polarization opponent axons from two neighboring ommatidia whose principal axes differ by ΔΣ=5, 20 and 40 deg. As ΔΣ increases, the amount of polarization state space sampled also increases. (F) Loci plotted in open black circles are for degree of polarization (DoP)=1.0 and angle of polarization ɸ ranging from −90 to 90 deg. Loci plotted with lines and symbols are at fixed ɸ and varying DoP ranging from 0 to 1. The neural connectivity diagram depicts our hypothesis for a robust polarization detector involving aligned and substantially misaligned adjacent ommatidia.

This method builds on the work of Nilsson and Howard (1989), who studied depolarizing properties of butterfly eyeshine by illuminating with linearly polarized white light and viewing eyeshine through an analyzing filter crossed with respect to the incident polarization. The analyzing filter blocks the rhabdom's dominant first-order waveguide modes when the angle of polarized illumination is either parallel or perpendicular to rhabdomeric microvilli, making it possible to view only depolarized higher-order modes. When they rotated the polarizer/analyzer combination by 30 or 60 deg, keeping the eye fixed, depolarized first-order modes were created owing to microvillar birefringence. We took a similar approach but kept the crossed polarizer/analyzer fixed and rotated the eye in 5 deg angular increments, taking an eyeshine image at every angular position θ (Fig. 3A).

The observed depolarized eyeshine is caused by microvillar birefringent interference. A linearly polarized light wave incident upon an ommatidium can be resolved into two components, one parallel to microvilli and the other perpendicular to them. These two components excite two waveguide modes propagating along the rhabdom. Because of the weak birefringence of the rhabdom, the two modes gradually fall out of phase as they undergo a round trip within the ommatidium. When exiting the eye, the two modes interfere and form an elliptically polarized light wave in free space (characterized by small ellipticity, defined as the ratio between the minor and major axes of the polarization ellipse). The part of this elliptically polarized light that transmits through the polarization analyzer (which is orthogonal to the polarizer that produces the incident illumination) thus contributes to the observed depolarized eyeshine. Therefore, by ‘depolarized light’ or ‘depolarization’, we mean linearly polarized eyeshine orthogonal to the initial linearly polarized incident light. The origin of depolarization is the birefringent rhabdom, and not phase scrambling as light propagates along the rhabdom (for example, as the result of irregularities in geometry or refractive indices of the rhabdom).

Depolarized eyeshine is strongest when the angle of polarization of the incident linearly polarized light is at 45 deg to the microvilli. When the eye is rotated such that the angle of polarization increases to 90 deg, the incident light is aligned with the microvillar axis and thus the intensity of depolarized eyeshine should decrease to zero. If we continue rotating the eye to 135 deg, the intensity should gradually increase to a maximum at 135 deg at about the same level as that observed at 45 deg. That is exactly what is observed experimentally (Fig. 3B). This is powerful evidence for all microvilli that contribute to the depolarization being precisely parallel or perpendicular to one another in a straight rhabdom with no twist or wobble. In the ommatidia of *J. evagoras*, red light is absorbed relatively less by all the rhodopsins and metarhodopsins; the observed depolarized eyeshine is therefore red in color.

All studied individuals of *J. evagoras* were reared from eggs on potted host plants grown from seeds of *Acacia melanoxylon* in a greenhouse in Cambridge, MA, USA. After eclosion, an adult was kept in a small net cage and fed daily with honey water. Before measurement, a butterfly was first quieted by refrigerating it for a few minutes at 5°C. To avoid eye damage, butterflies within the first few days of eclosion were used. A soft wax was used to fasten the wings together at the base in the closed resting position, and an individual was held stationary in a slotted hollow tube with the fastened wings in the slot and the head extending halfway out of the end of the tube. If the right eye was chosen for measurement, the back of the left side of the head was fastened to the tube with wax, with its palps also gently held to the side with wax, altogether stabilizing the head.

The prepared butterfly was fixed to a multi-axis Leitz (Oberkochen, Germany) UT-4 goniometer stage, and then set under a Wild (Eppelheim, Germany) M7 stereo microscope equipped with incident-light prism. The orientation of the tube was adjusted so that one stage axis controlled body pitch (elevation viewed), and another controlled body yaw (azimuth viewed). The point at the center of the acute zone, where the illuminated eye patch is largest, is defined as elevation 0 deg, and the point where both eyeshine spots on each eye are symmetrical with respect to the head is defined as azimuth 0 deg. The stage axes are then adjusted to set the viewing direction to be measured. Eye patches of 21 deg were measured at elevations of −30, 0, 30 and 60 deg, which were located at an azimuth of 10 deg with a few patches at an azimuth of 15 deg.

The goniometer was then moved to the calibrated circular stage of a Leitz Ortholux-Pol microscope, equipped with ×8/0.18P objective and ×16 eyepieces. The epi-illumination was supplied by a halogen heat-filtered, neutral density (ND) filtered, linearly polarized white light equipped with a Vincent Uniblitz shutter (Vincent Associates, Rochester, NY, USA). The polarization analyzer in the body of the microscope was set to ‘crossed’ with respect to the incident polarization, and then moved out of view.

We focused the microscope on the eye and then inserted a ND 2.0 filter and switched on the epi-illumination. After finding the eyeshine patch, we focused inward until the patch collapsed to a bright spot, and then adjusted the goniometer position until there was no wobble of the eyeshine spot with rotation. We then focused outward to the corneal facets. We set θ, the orientation of the circular stage, so that dorsal was at the top of the image viewed, thereby defining θ**=**0 deg.

After these adjustments, the corneal facets were Köhler illuminated by the 21 deg conical beam from the ×8/0.18P microscope objective. Thus, every ommatidium in the illuminated patch at the front focal plane was illuminated by the 21 deg conical beam. So, any ommatidium that is within the illuminated patch and has its optical axis within the 21 deg beam exhibits depolarized eyeshine of intensity that depends on the difference in angle between the AoP of the illumination and the microvilli of the rhabdom of that ommatidium.

We used an Olympus (Shinjuku, Kanto, Japan) TG-1 digital camera fixed to the left eyepiece, rotated 90 deg clockwise to fit between the eyepieces, set to the Custom program in Macro mode, with 8Mp, ‘fine’ Jpeg compression, Incandescent WB, Spot AF mode, ESP metering and 4.0 Zoom.

We switched off the epi-illumination, waited for several minutes, and then replaced the ND 2.0 filter with ND 1.5 and took a trial photo at ISO 800 by manually switching on the light and simultaneously depressing the shutter, holding both for 1 s, and then releasing both. If the photo was too dim or too bright, we adjusted the ND filter accordingly. Next, we increased ISO to 3200 and moved the polarization analyzer into the path of reflected light. This allows a photo to capture only depolarized eyeshine. A series of 37 photos was taken (θ**=**0 deg through 180 deg) by rotating the circular stage 5 deg after each photo, waiting at least 1 min between photos. For photo controls, we rotated back to θ**=**0 deg and took a photo, and then another with reduced ISO and analyzer removed. Comparison of the control photos with initial photos allows evaluation of bleaching caused by the photo series.

All photos were rotated 90 deg counter-clockwise to compensate for the rotated orientation of the camera, then processed in ImageJ (Schneider et al., 2012, 2012), grouping images into image stacks that were aligned using the ‘StackReg’ ImageJ plugin with a Rigid Body transformation (Thévenaz et al., 1998). An arbitrary hexagonal coordinate system was created for each patch, assigning a unique coordinate pair to each ommatidium in each stack of images. These stacks were then separated into RGB layers, creating separate R, G and B stacks. Intensities of red, green and blue depolarized eyeshine were then measured for each ommatidium in these stacks using the Microarray Profile plugin (http://imagej.nih.gov/ij/), which uses a grid of circular regions of interest (ROIs) to measure the intensity values of each ommatidium in a stack of photos simultaneously.

### Polarization model description

In the eyes of decapods, which have provided detailed model systems for the study of polarization vision, microvilli of all rhabdoms in the ommatidial array are oriented in one of two orthogonal directions, and this alignment is constant throughout the entire length of each rhabdom (Waterman, 1981). Thus, depolarization versus θ of all ommatidia should wax and wane together, in phase. However, this is not the case for the ommatidial patch of *J. evagoras*, as shown in Fig. 3A. Individual ommatidia exhibit a range of depolarization versus θ responses. An example for two adjacent ommatidia is shown in Fig. 3B. The deep local minima in the responses of both ommatidia are separated by 90 deg with local maxima halfway between the two minima. This deep, regular modulation implies that microvilli contributing depolarization do not twist or wobble: the rhabdoms of both ommatidia are straight, just as in decapods. Although the responses of the two adjacent ommatidia in Fig. 3B have the same shape, they are shifted in phase by 30 deg. One ommatidium is simply misaligned by 30 deg with respect to its neighbor.

Surprisingly, we found that many rhabdoms in the measured patch were substantially misaligned (Fig. 3C,D). We characterized this misalignment by computing the distribution function of ΔΣ, the difference in alignment of adjacent ommatidia (Fig. 3D): 22% were aligned exactly, 50% were aligned within ±5 deg, 30% were misaligned by 15 deg or more, and 15% were misaligned by 20 deg or more.

Processes of edge detection and motion detection require aligned ommatidia and should involve no misaligned ommatidia. How might the misaligned ommatidia be useful for vision? We note that having first-order opponent interneurons polarization blind at an AoP of 45 deg poses a serious challenge for butterflies. However, if a second-order neuron simply sums first-order outputs from a pair of aligned and substantially misaligned adjacent ommatidia, its response versus AoP is robust, and is never polarization blind. Thus, we present a theoretical model of polarization vision in which second-order polarizational interneurons function as robust polarization detectors.

To decide on conservative criteria for identifying aligned ommatidia in the array that are suitable for ED and pairs of adjacent ommatidia suitable for robust PD, we compared responses from simulated first-order opponent interneurons from an adjacent pair of ommatidia for receptors of polarization sensitivity=10, for linearly polarized light with ɸ ranging from −90 deg to 90 deg, and for DoP***=***1 (Bernard and Wehner, 1977). Then, we plotted the opponent responses of one ommatidium versus opponent responses of its neighbor in a polarization state space (Fig. 3E). The responses of the pair with ΔΣ**=**5 deg differ little from strict proportionality characteristic of an aligned pair. To focus on sub-populations of ommatidia most likely to underpin detection of edges, we therefore conservatively define ‘aligned ommatidia’, suitable for edge detection and motion detection (ED), to be linear groups of three or more ommatidia that deviate from parallel by no more than ±5 deg. Similarly, we conservatively define ΔΣ≥20 deg to be ‘large ΔΣ’, suitable for robust PD, performed by a second-order neuron that simply sums its two first-order inputs, as suggested by the neural diagram of Fig. 3F. The response of this robust second-order PD neuron at all values of ɸ is substantial if ΔΣ is 20 deg or larger. It is never polarization blind.

### Statistical methods and analysis

#### Polarization discrimination behavioral experiment

Preferences of free-flying, patrolling individuals of *J. evagoras* to approach robots with different wing treatments were recorded in 20 min trials for each of the three color/polarization treatments, and measured in 6–7 separate geographic field sites each, with each site consisting of patches of acacias occupied by larvae and pupae of *J. evagoras*. Specifically, ‘3M+Red G280’ was tested in 10 trials (20 min each) carried out at six sites, ‘3M+Yellow R312’ was tested in six trials at six different sites, ‘3M+Blue R374’ was tested in 10 trials at 7 different sites. Sites were spaced no closer than 300 m apart, with the farthest sites separated by over 40 km, to reduce the likelihood that individuals encountered at one site would also be present in subsequent trials at nearby sites. To further avoid the possibility of counting responses by the same individual butterflies more than once, we analyzed these results using site as the unit of replication, summing the total number of responses to polarized and depolarized models at each site for each treatment, and calculating one-sample Student's *t*-tests for the difference from zero of these total differences between polarized and depolarized responses across sites for each treatment.

#### Wing reflectance and perceptual analysis

Spectroscopic reflectance measurements were conducted for the dorsal forewings and hindwings of 12 female and 13 male *J. evagoras* pinned museum specimens. The mean and s.e.m. reflectance values were calculated and plotted. We used R package pavo (Maia et al., 2019) to generate coordinates in tetrahedral color space (Stoddard and Prum, 2008) for each of the collected reflectance spectra. A tetrahedral color space is a chromaticity diagram that represents the gamut of all perceivable colors given a set of curves representing the sensitivity of the four photoreceptors of a tetrachromatic organism. Using the tetrahedral color space, we can then calculate coordinates that represent the perceived color of any given reflectance spectrum. We used sensitivity curves for each of the four photoreceptor types found in *J. evagoras* which indicated sensitivity from 300 to 700 nm (Fig. S2). Because reflectance spectra were recorded only from 350 nm, we truncated the raw sensitivity curves to 350–700 nm. As we were primarily interested in the color of the wings themselves, rather than color in a particular context, we also assumed ideal lighting and background color (Maia et al., 2019). Coordinates in tetrahedral color space can be defined in a number of ways: cone stimulations (absolute or relative values denoting estimated stimulation of each of the four photoreceptor types), hue and saturation (hue is denoted by azimuth and elevation of the color relative to the center of the tetrahedron, and saturation indicates the length of the vector from the center of the tetrahedron to the color), and finally the Cartesian coordinates (*x*, *y*, *z*) of the color in the tetrahedral color space. Here, we elected to use Cartesian coordinates, because they have better statistical properties than the other measures (relative cone catches sum by definition to unity so are not independent, and the circular coordinate system for hue and saturation result in statistical complications). To test whether color significantly differed between males and females, we used the RRPP package in R (Collyer and Adams, 2018), using multivariate response linear modeling of the effect of sex on the Cartesian coordinates of each color in tetrahedral color space, as well as on the raw spectral data, for comparison. *P*-values from this combined analysis were corrected for multiple comparisons via the method of Benjamini and Hochberg (1995).

In addition, we summarized color contrasts between reflectance measurements of museum specimens and robot wing models. These contrasts were generated by computing the (unweighted) Euclidean distance between colors in the tetrahedral color space. Colors that are perceptually more similar will generally have lower Euclidean distances. We chose this measure, rather than weighted Euclidean distances (just-noticeable differences, JNDs; Vorobyev and Osorio, 1998; Maia et al., 2013) because we do not have good estimates for the required parameters (Weber fraction, photoreceptor densities) for *J. evagoras*.

#### Degree of polarization

We also measured the polarization content of reflected light at both λ=360 nm and λ=450 nm. We computed the DoP of male and female specimens as well as of the mock wings, defined as |*I*_H_−*I*_V_|/(*I*_H_+*I*_V_), with *I*_H_ and *I*_V_ calculated by taking the total intensity of a polarization image, at a range of angles of incident light θ and detection angles ϕ. We analyzed DoP as a function of the combined angle between θ and ϕ **(**which we termed ‘angular contrast’ and can be thought of as the obliquity of the viewing angle with respect to the source of illumination). The relationship between angular contrast and DoP in blue and UV appeared to be non-linear, so we tested various linear and polynomial mixed-effects models that analyzed the relationship between these two variables and sex. The relationship between DoP in UV and angular contrast was best described by a second-order polynomial (Table S1), whereas for DoP in blue, a third-order polynomial provided the best fit (Table S1). The significance of the orthogonal polynomial terms of angular contrast, sex and their interactions were evaluated using parametric bootstrapping and model comparison. First, a maximal mixed-effects linear model was fitted that considered sex, the orthogonal polynomial terms of angular contrast, and their first-order interactions as fixed effect terms, accounting for repeated measurement of individuals by fitting random intercepts for individuals. Then, to calculate the significance of each of these fixed effect terms, ‘smaller’ models were constructed that lacked each of these terms and were compared via parametric bootstrapping against ‘larger’ models that included these terms (but no higher order terms). Effect subtraction proceeded hierarchically from interaction to individual fixed effects. Larger and smaller models compared in this way thus differ only by the term of interest.

The maximal model, containing all of the fixed effects and their interactions, described above, was the best fit, most parsimonious model, and therefore was selected to generate model fits and confidence bands using the ‘effects’ package in R (Halekoh and Højsgaard, 2014; Fox, 2003). Confidence bands, where included, denote smoothed means and standard errors. Parametric bootstrapping was done using the ‘Pbmodcomp’ function of the ‘pbkrtest’ package in R. All mixed-effects models were fitted using maximum likelihood with the ‘lmer’ function of package ‘lme4’ (Bates et al., 2015) in R (version 3.4.1, http://www.R-project.org/). *R*^2^ values for testing model fits were obtained using the r.squaredGLMM function of the ‘MuMIn’ package (https://CRAN.R-project.org/package=MuMIn) and the R2 function in the semEff package (https://CRAN.R-project.org/package=semEff).

#### Analysis of the effects of sex and elevation on ED and PD

The effects of sex and elevation (along the ‘forward-facing’ part of the eye, azimuth of ∼10 deg) within the eye on the percentage of putative edge detectors and polarization detectors (%ED and %PD ommatidia, as well as the more specific ‘%ED-only ommatidia’ and ‘%PD-only ommatidia’ metrics; see Results for metric definitions) were investigated using mixed-effects linear models followed by parametric bootstrapping and model comparison in a similar fashion to the polynomial mixed-effects model workflow above.

The maximal model, that considered sex, elevation, and their first-order interaction as fixed effect terms, accounting for repeated measurement of individuals by fitting random intercepts for individuals within the effect of elevation, was the best fit, most parsimonious model, and therefore was selected to generate model fits and confidence bands using the ‘effects’ package in R (Fox, 2003). Confidence bands, where included, were generated using the Kenward–Roger coefficient–covariance matrix to compute effect standard errors, as implemented in the ‘effects’ package. Parametric bootstrapping was done using the ‘Pbmodcomp’ function of the ‘pbkrtest’ package in R (Halekoh and Højsgaard, 2014). All mixed-effects models were fitted using maximum likelihood with the ‘lmer’ function of the package ‘lme4’ (Bates et al., 2015) in R (version 3.4.1, http://www.R-project.org/). Plots were made using the ggplot2 package in R (https://CRAN.R-project.org/package=ggplot2), and manually edited in Inkscape (version 0.92.4, Inkscape Project).

### Ommatidial histology

*Jalmenus evagoras* eyes were dissected under a stereomicroscope at room temperature (RT) and immersed in fixative solution (2.5% formaldehyde, 2.5% glutaraldehyde in 0.1 mol l^−1^ cacodylate buffer pH 7.4, Electron Microscopy Sciences, Hatfield, PA, USA) for 1 h at RT then stored overnight at 4°C. Subsequently, the perfused fixed eye tissue was washed with 0.1 mol l^−1^ cacodylate buffer, post-fixed for 1 h with 1% osmium tetroxide (OsO_4_) containing 1.5% potassium ferrocyanide (KFeCN_6_), washed in water 3 times and incubated in 1% aqueous uranyl acetate for 1 h. After two additional washes with water, the tissue was dehydrated for 10 min in increasing grades of alcohol (50%, 70%, 90%, 100%), placed in propylene oxide for 1 h, and infiltrated overnight in a 1:1 mixture of propylene oxide and TAAB Epon. The tissue was then embedded in TAAB Epon for polymerization at 60°C for 48 h. The eye tissue was excised with surrounding solid Epon, precisely realigned to 30 deg elevation in the dorsal region, prior to a second resin polymerization step.

Ultrathin 60 nm sections were cut with a diamond blade on a Reichert Ultracut-S microtome at the Harvard Medical School Electron Microscopy Facility and sections at recorded depths were placed onto copper grids. To increase contrast prior to examination with the electron microscope, the copper grids were stained at RT for 30 min with 2% uranyl acetate then for 4 min with Reynold's lead citrate. Images were obtained using a JEOL JEM 1400 Plus Transmission electron microscope (TEM) at the Lund Microscopy Facility. The principal rhabdomeric axes for contributing photoreceptor cells (R1-R8) were recorded in ommatidial arrays of 10–15 individual visual units across depth from the upper rhabdomeric tier to the equatorial zone.

## RESULTS

### Adult *J. evagoras* can utilize polarization signals under field conditions

**Fig. 4.**
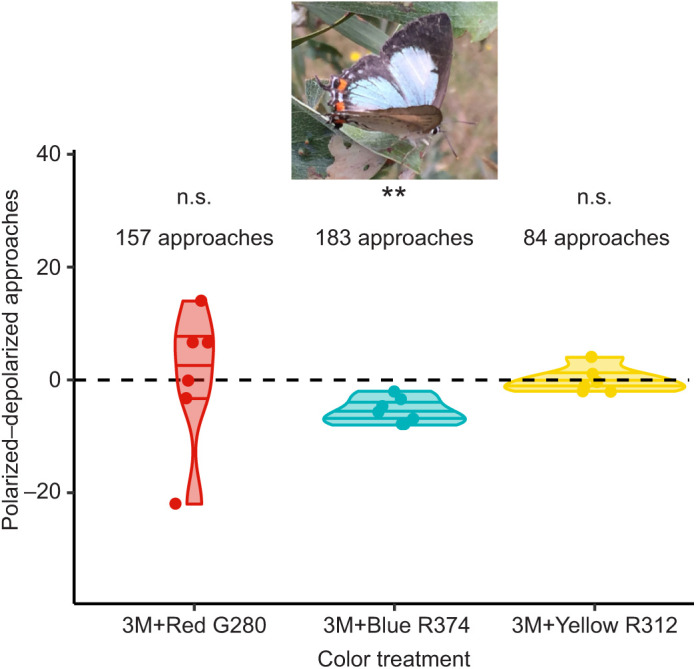
**Difference between preferences of flying *J. evagoras* individuals to approach and inspect polarized and depolarized wing robot models.** Butterflies preferentially approached the ‘blue’ (3M+Blue R374) wing beating robot that contained disrupted polarization content (Student’s *t*-test, *t*=−6.2185, **P=0.0008 for polarized−depolarized=0 at *N*=7 sites, 183 responses total), compared with those that had strong linear polarization. However, they showed no preference (Student’s *t*-test; n.s., not significant) for either ‘red’ (3M+Red G280, 157 responses total) or ‘yellow’ (3M+Yellow R312, 84 responses total) wing models (see Materials and Methods for additional specific color information). Points are the total number of polarized approaches minus the total number of depolarized approaches for each site, with zero denoted by the dashed horizontal line. Variability along the *x*-axis is random ‘jitter’ introduced to help separate points for visualization.

A total of 424 responses of free-flying, patrolling individuals of *J. evagoras* to our robot wing treatments were recorded in 40 separate 20 min trials for three color/polarization treatments measured in each of 6–7 separate geographic field sites, each consisting of patches of acacias occupied by colonies of larvae and pupae of *J. evagoras*. Individuals preferentially approached robot models that contained disrupted blue (‘3M+Blue R374’) polarization content compared with those that had strong linear blue polarization (i.e. values less than 0 in Fig. 4), where 113 (61%) of 183 total approaches were to depolarized models (*t*=−6.2185, *P*=0.0008 for polarized–depolarized=0 at *N*=7 sites; Fig. 4). Approaches to ‘3M+Yellow R312’ and ‘3M+Red G280’ wing models (see Materials and Methods for specific color information) did not vary by polarization content, with 42/84 approaches to depolarized (50%) and 77/157 approaches to depolarized (49%) (not significant; Fig. 4), respectively.

### *Jalmenus evagoras* exhibits sexual dimorphism in wing color and DoP

**Fig. 5.**
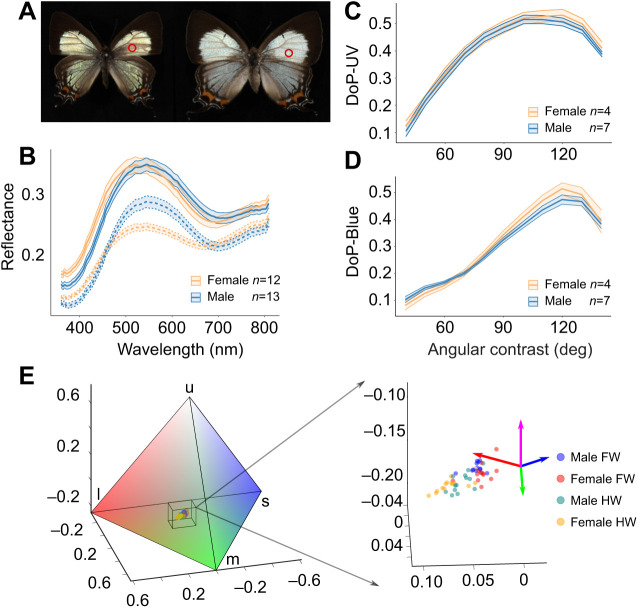
**Sexual dimorphism in *J. evagoras*: reflectance, color and degree of polarization.** (A) Male (left) and female (right) specimen images of *J. evagoras*. Red circles indicate the approximate patches measured for spectral reflectance. (B) Reflectance spectra of *J. evagoras* wings for 12 females and 13 males. Spectra were measured on forewings (solid lines) and hindwings (dashed lines) at normal incidence (θ=0) and an oblique detection angle (ϕ=55 deg). (C) Degree of polarization (DoP) in UV (λ=360 nm), as a function of θ+ϕ (‘angular contrast’) for male and female butterflies. Here, θ ranges from −60 to 70 deg and ϕ from 10 to 70 deg, both in steps of 10 deg. DoP is defined as abs[(*I*_H_−*I*_V_)/(*I*_H_+*I*_V_)], with *I*_H_ and *I*_V_ calculated by integrating the intensity of the butterfly images in Fig. S1 in the polarization photos at certain θ and ϕ. (D) DoP in blue (λ=450 nm) as a function of angular contrast for male and female butterflies. The largest DoP is achieved at angular contrasts of around 120 deg and is slightly larger for the female butterfly under both UV and blue illumination. In B–D, the means and standard error ribbons are plotted for illustration purposes. (E) *Jalmenus evagoras* wings in tetrahedral color space. The tetrahedral color space represents the relative stimulation of each of the four rhodopsins indicated by the labeled vertexes: ‘u’ for UV, ‘s’ for short wavelength (blue), ‘m’ for medium wavelength (green) and ‘l’ for long wavelength (red) (see Materials and Methods). Points locate individual specimen wing spectra in the color space, and the bounding box denotes locations of specimen spectra in overall color space. Color coordinates of specimen spectra are shown on an expanded scale on the right to show differences between males and females for the forewings (FW) and hindwings (HW); colored arrows indicate the direction of the tetrahedron's vertexes, with magenta indicating UV.

On the dorsal side, the wings of *J. evagoras* appear a shimmery light blue-green, except for the submarginal region, which is black (Fig. 5A). Spectroscopic measurements and polarization imaging were conducted for the dorsal side of the wing specimens. The results show subtle but important differences in both coloration and polarization properties between male and female wing specimens. Male and female forewings are similarly reflective, but the reflectance spectra of females are blue shifted, peaking at λ≈521 nm compared with λ≈545 nm for male forewings (Fig. 5B). In contrast, male hindwings were observed to be brighter than those of females despite having similar peak wavelength values.

We next generated coordinates in tetrahedral color space (Stoddard and Prum, 2008) using the R package ‘pavo’ (Maia et al., 2013) and the four *J. evagoras* opsin sensitivity curves (see Fig. S2), for each of the collected reflectance spectra. To aid in conceptualizing the tetrahedral color space, we generated an overall view of the *J. evagoras* tetrahedral color space (Fig. 5E), which showed that the measured spectra were relatively desaturated. Enlarging this area showed that males and females inhabit adjacent but largely non-overlapping regions of the tetrahedral color space (Fig. 5E right). To determine whether males were significantly spectrally different from females, we used the RRPP package in R to analyze the effect of specimen sex on two multivariate response datasets: the spectral reflectance data as a whole and the Cartesian coordinates of specimens in tetrahedral color space using multivariate response linear modeling. With the high number of variables (wavelengths) in the raw data, there was insufficient power to resolve the differences between males and females in *J. evagoras.* However, when these were plotted in tetrahedral color space, we found the effect of sex was highly significant for both forewings and hindwings (*F*=12.51, adjusted *P*=0.002 for forewings, *F*=36.28, adjusted *P*=0.0004 for hindwings).

**Fig. 6.**
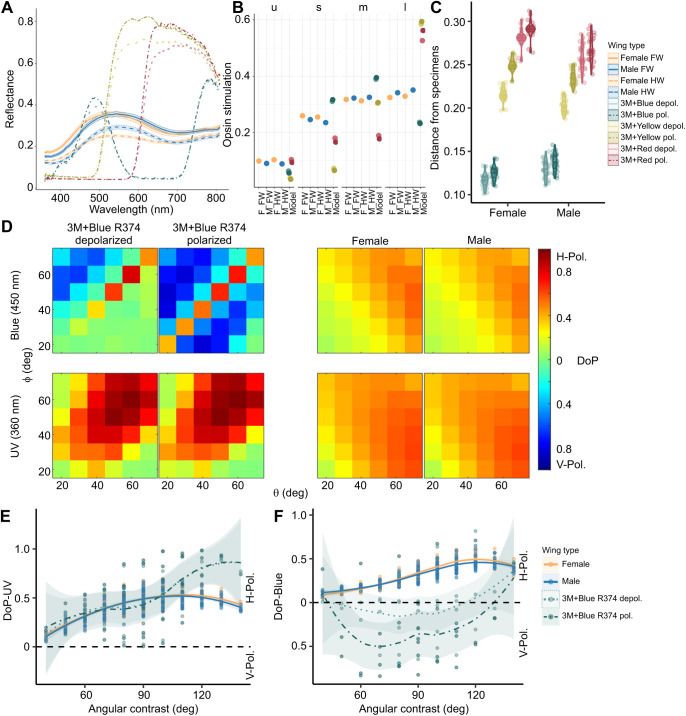
**Spectral and polarization properties of butterfly wing models and specimens.** (A) Spectral reflectance properties of constructed butterfly wing models (dot-dash lines without confidence bands) employed in the behavioral experiment, with the spectra of the pinned butterfly specimens (lines with confidence bands) overlaid for comparison. Error bands on specimen spectra represent the s.e.m. (B) Dot-plot showing the relative stimulation values of the wing models and the mean stimulation values of the *J. evagoras* specimens (F, female; M, male; FW, forewing; HW, hindwing) for the four rhodopsins: ‘u’ for UV, ‘s’ for ‘short wavelength’ (blue), ‘m’ for ‘medium wavelength’ (green) and ‘l’ for ‘long wavelength’ (red) (see Materials and Methods). (C) Violin plot of unweighted Euclidean distances in tetrahedral color space between the reflectance spectra of pinned male and female butterfly specimens from Fig. 5 and polarized and depolarized wing models. Lower Euclidean distances indicate more similar colors. (D) Heatmaps of horizontal (H) or vertical (V) DoP of 3M+Blue R374 polarized and depolarized wing models and biological specimens under both blue (450 nm) and UV (360 nm) light across a range of detection (ɸ) and incident (θ) light angles. (E) Horizontal or vertical DoP of 3M+Blue R374 wing models as a function of total angular contrast (detection angle+incident light angle) under UV (360 nm) light. (F) Horizontal or vertical DoP of 3M+Blue R374 wing models as a function of total angular contrast under blue (450 nm) light. Male and female specimen DoP traces are included in E and F as a reference. As in Fig. 5, solid lines denote forewings, and dashed lines denote hindwings. Polarized wing model traces are denoted by dot-dash lines and ‘depolarized’ wing model traces are denoted by dotted lines. In E and F, the smoothed means with loess confidence ribbons are plotted for illustration purposes.

In addition, color contrasts between reflectance measurements of museum specimens and constructed wing models show that both males and females (across both wing types) were most similar to blue wing models, followed by yellow and red DBEF-Q color models, successively (Fig. 6C).

Following evidence that *J. evagoras* butterflies have large amounts of blue-absorbing rhodopsins in their acute zones as well as high polarization sensitivity to UV and blue polarized light (Fig. S2), we also measured the polarization content of reflected light at both λ=360 nm and λ=450 nm. For both sexes at both wavelengths, the H-polarized (perpendicular) component in the reflected light (*I*_H_) was much stronger than the V-polarized (parallel) component (*I*_V_, by a factor as large as two, data not shown). We computed the DoP of male and female specimens, defined as (*I*_H_−*I*_V_)/(*I*_H_+*I*_V_), with *I*_H_ and *I*_V_ calculated by taking the total intensity of a polarization image, at a range of angles of incident light θ and detection angles ϕ. We found that at both λ=360 and λ=450 nm, when the combined angle between θ and ϕ **(**which we termed ‘angular contrast’) approached 120 deg, female specimens had a larger DoP than male specimens. The relationship between angular contrast and DoP in blue and UV appeared to be non-linear, so we tested various linear and polynomial models that analyzed the relationship between these two variables. The relationship between DoP in UV and angular contrast was best described by a second-order polynomial (Table S1), whereas for DoP in blue, a third-order polynomial provided the best fit (Table S1). The significance of the orthogonal polynomial terms of angular contrast, sex and their interactions were evaluated using parametric bootstrapping and model comparison. We found that all orders of polynomial angular contrast were significant in predicting DoP in both UV and blue (Table S1), but that only for DoP in blue were the interactions of sex and polynomial contrast significant (sex×angular contrast first-order: *t*=11.45, *P*=0.0008; sex×angular contrast second-order: *t*=4.44, *P*=0.0372; Table S1).

### Behaviorally preferred wing models vary in both spectral and polarization properties

After quantifying the spectral and polarization properties of male and female *J. evagoras* specimens, we conducted the same analysis on the wing models used in the behavioral experiment and compared the results against those we obtained from the butterfly specimens. We first analyzed the spectral reflectance properties of the wing models using the same technique employed for the specimens (Fig. 6A). We quantified the relative opsin stimulation values (Fig. 6B) and unweighted Euclidean distance in tetrahedral color space between the reflectance spectra of the wing models and the specimens (Fig. 6C) and found that both male and female specimens were closest in perceptual color space to the blue wing models, followed by the yellow and red models. We next analyzed the polarization properties of blue wing models under blue (450 nm) and UV (360 nm) illumination. We focused on this wing model because it elicited the strongest approach responses from butterflies in the field (Fig. 6D). As with the specimens, we analyzed the reflectance of H- and V-polarized light to find the DoP across a range of angles of incident light θ and detection angles ϕ (Fig. 6D). Under blue light, blue wing models were primarily V-polarized, while the butterfly specimens were primarily H-polarized. However, the blue polarized wing models also exhibited very strong H-polarized specular reflection compared with their depolarized counterparts (when detection and incident angles are identical) from the multiple reflective interfaces used to construct the wing models (Fig. 6D). Under UV light, blue polarized wing models were highly H-polarized. Under both kinds of illumination, blue depolarized wing models exhibited a lower degree of polarization than the polarized counterpart (Fig. 6D). We also compared the DoP of these wing models and the specimens together, in relation to the total angular contrast (the sum of the incident and detection angles; Fig. 6E,F). Under UV light, both polarized and depolarized wing models exhibited H- polarization with highly variable DoP that overlapped substantially with the DoP of both male and female specimens across a wide range of angles (Fig. 6E). However, under blue light, despite substantial variability across angles, the depolarized blue model DoP, which is largely unpolarized, was more similar to that of the butterfly specimens than the polarized blue, which exhibited strong V-polarization (Fig. 6F).

### Many ommatidia in the array are misaligned

To learn how microvilli are organized within rhabdoms of individual ommatidia, we measured the variation in depolarized ommatidial eyeshine intensity as a function of eye rotation (roll angle θ). If the AoP of illumination is either parallel or perpendicular to rhabdomeric microvilli, there is no depolarization. However, if the AoP is oblique, say 45 deg, there is substantial depolarization caused by microvillar birefringent interference. The AoP vector of the linearly polarized illumination can be resolved into two components, one parallel to the microvilli and the other perpendicular to them. Birefringence means that the propagation constants of those two components are different, so as the two mutually perpendicular, excited waveguide modes propagate down the rhabdom they fall out of step and interfere, thus creating depolarization. At some (unknown) depth, interference creates wideband depolarized light, some of which propagates back up the rhabdom and out of the eye after being partially absorbed by rhodopsins and metarhodopsins, red depolarization suffering much less absorption than blue or green components. Consider what should happen when the eye is rotated and the AoP increases to 90 deg: the intensity of red depolarized eyeshine from a single ommatidium should decrease gradually to zero at 90 deg. If we continue rotating the eye to 135 deg, the intensity should gradually increase to a maximum at 135 deg at about the same level as observed at 45 deg, which is exactly what was observed experimentally (Fig. 3B). This is powerful evidence for all microvilli that contribute to the depolarization from a single rhabdom being precisely parallel or perpendicular to one another.

We define the microvillar principal axis (Σ) as the angle at which the depolarized eyeshine is minimal. It repeats every 90 deg (Mod 90 deg), so we chose Σ in the range 0 to 90 deg. The two adjacent ommatidia of Fig. 3B have the same shape as described above but are misaligned by 30 deg.

**Fig. 7.**
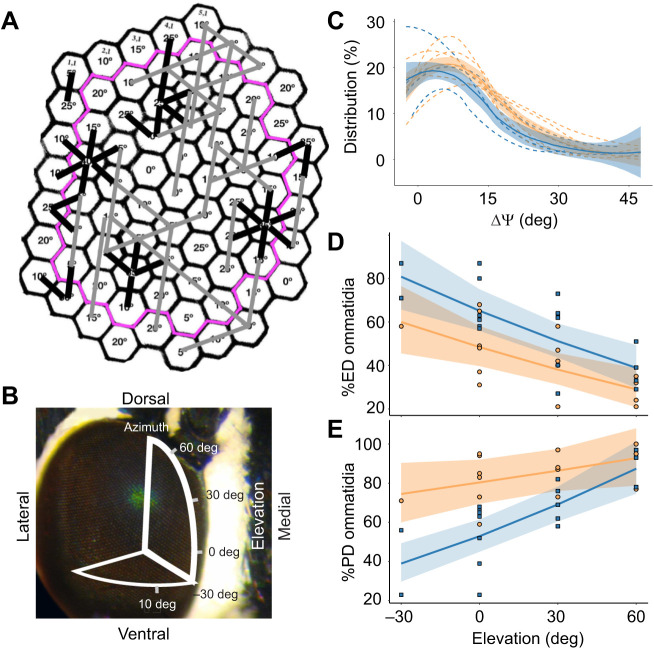
**Conservative theoretical exercise to explore the design of edge detector and polarization detector subarrays.** (A) Ommatidial array of Fig. 3 annotated with principal axes Σ. Our conservative theoretical exercise defines edge detectors as rows of three or more ommatidia along which Σ deviates no more than ±5 deg (marked here with straight gray bars), and defines robust polarization detectors as misaligned adjacent pairs of ommatidia for which ΔΣ is at least 20 deg (marked with short black bars). The pink outline encloses all ommatidia that have six measured nearest neighbors (*N*=48 interior ommatidia), from which final values were calculated. (B) *Jalmenus evagoras* compound eye, overlaid with a diagram showing the elevational angles sampled across the eye. (C) Microvillar principal axes Σ are heterogeneous across all sampled elevational patches, and most ommatidia are misaligned at least 10 deg with respect to their nearest neighbors. The distribution of ΔΣ between neighboring ommatidia of patches at 0 deg elevation is plotted as separate, smoothed, dashed lines for each individual, while the solid lines with standard error bands show the average distribution and standard error of ΔΣ for males and females collectively. At other elevations, ΔΣ distributions are shifted towards larger angles. Smoothed lines were calculated using locally weighted regression as employed by the loess method in the geom_smooth function of the ggplot2 package. (D) The percentage of ommatidia that are suitable for edge detection (ED) declines significantly with increasing elevation in the eye (*T*=23.056, *P*=0.0001). Males show a significantly higher overall percentage of such ommatidia at all elevations (*T*=6.882, *P*=0.0183). (E) The percentage of adjacent pairs of ommatidia that are suitable for robust polarization detection (PD) increases significantly with increasing elevation in the eye (*T*=19.885, *P*=0.0001). Overall, females exhibited a significantly larger percentage of PD ommatidia than males (*T*=7.977, *P*=0.012). In C–E, males are plotted in blue and females are plotted in orange. In D and E, the smoothed conditional means are plotted for the interaction effect of sex and elevation from our mixed-effects linear model analysis, with 95% confidence bands plotted for illustration purposes, calculated using the Kenward–Rogers approximation for fixed-effect standard error (Fox, 2003).

Many neighboring ommatidia are misaligned with respect to one another, with differences in principal axes of adjacent pairs ranging up to 45 deg. We used this method to characterize patches of ommatidia (as in Fig. 7A) at four elevations of view (Fig. 7B) for 6 male and 7 female *J. evagoras*. We found that all studied patches exhibited considerable heterogeneity in rhabdomeric alignment (Σ; Fig. 7C). Additionally, we conducted a histological examination under a TEM microscope of individual ommatidia of *J. evagoras* males in the dorsal eye at 30 deg elevation, where our *in vivo* analyses revealed many misaligned ommatidia. We found that the rhabdomeric microvilli of neighboring ommatidia, similar to *in vivo* eyeshine evidence, exhibit a wide diversity of microvillar orientations (Fig. S3). We are not the first to demonstrate misaligned ommatidia in butterflies: see Labhart et al. (2009) for the dorsal rim of the monarch eye.

As described in Materials and Methods, our conservative theoretical model for ommatidia suitable for ED involves linear groups of three or more ommatidia that are aligned within ±5 deg. These groups of ‘ED’ ommatidia are marked in the example in Fig. 7A with long gray bars. Our theoretical model for robust PD involves pairs of adjacent ommatidia that are misaligned by 20 deg or more. These pairs of ‘PD’ ommatidia are marked in Fig. 7A with short black bars.

Spatial vision of butterflies necessarily involves temporal scanning. Behaving butterflies are in constant motion, spending very little time fixating (see Movies 1 and 2). Their ommatidial arrays are inhomogeneous, composed of a mixture of ommatidia having different organization of rhabdomeres within their rhabdoms, so eye movements are necessary for them to build a representation of what they need to see. This is true of other flying insects as well. Honeybees flying close to patterns do not require fixation, and can learn patterns and later recognize them regardless of whether the test patterns are stationary or in motion (Srinivasan, 1994).

We utilized mixed-effects linear models to investigate the relationship between these ED and PD metrics, patch elevation, and the sex of the studied individual. In both males and females, the percentage of PD ommatidia increased significantly with increasing patch elevation (*t*=19.885, *P*=0.0001; Fig. 7E), while ED sharply decreased (*t*=23.056, *P*=0.0001; Fig. 7D). These metrics also varied with sex, as females exhibited more PD (*t*=7.977, *P*=0.012) and less ED (*t*=6.882, *P*=0.0183) at all elevations, relative to males.

We also observed that these ommatidial arrays exhibited considerable variation in the organization of PD and ED ommatidia. While some groups of ommatidia within the patch were largely involved in both PD and ED with different neighbors, other groups were exclusively either one or the other, which we termed ‘PD-only’ or ‘ED-only’ ommatidia. We similarly studied the relationship between these more specific metrics and patch elevation or sex using linear models and found that both of these metrics demonstrated similar (and similarly significant) patterns to the more general PD and ED metrics (Fig. S4).

## DISCUSSION

The results of our field study indicate that free-flying individuals of *J. evagoras* butterflies can distinguish and respond to polarization signals, and that whether they do so varies for polarization at different wavelengths, exhibiting small, but unanimously consistent responses across sites and trials to blue polarized stimuli but not those of other colors. This may have been the result of the strong V-polarization in blue light present in the blue polarized wing models, which was largely negated in the depolarized wing models (Fig. 6F). Although more research is needed to fully disentangle the response of *J. evagoras* to the conflated signals of color, degree and angle of polarization, our results suggest that adults of *J. evagoras* are sensitive to either or both the degree and angle of polarization of visual stimuli under ecologically relevant field conditions.

These observed behavioral preferences are likely to be related to visual and morphological sexual dimorphism in this species. In agreement with qualitative field observations, the forewings of females are significantly blue-shifted (Fig. 5B) and exhibit a higher degree of H-polarization in blue than those of males (Fig. 5C,D), especially under oblique viewing angles with respect to incident lighting, such as might occur during face-to-face conspecific interactions under morning or afternoon sunlight. This aligns well with the retinal densitometry and polarization sensitivity data we have collected from several individuals, which suggest that the acute ommatidial zone of male and female *J. evagoras* has high amounts of blue and UV rhodopsins and exhibits the strongest overall polarization sensitivity to UV and blue light, though their polarization sensitivity is higher for UV, curiously (Fig. S2).

Conflicting requirements on the alignment of principal axes between adjacent ommatidia, necessary for ED and robust PD, forces a tradeoff. Individuals of *J. evagoras* are clearly receptive to polarized light, but how are their ommatidial sub-arrays designed so that both PD and ED can function? We observed two qualitatively different kinds of sub-array designs: (1) discrete groups of ommatidia within which most serve only one of those two functions, and (2) interdigitated mixtures where many ommatidia serve both functions. Indeed, we observed that dorsal eye regions contain a large fraction of misaligned adjacent ommatidia suitable for robust PD, but a small number of aligned ommatidia suitable for ED. As elevation decreased, we observed larger fractions of aligned ommatidia at the expense of fewer pairs suited to PD. Thus, as in other species of insects, the dorsal patches are particularly well suited to polarization vision, whereas equatorial regions of the eye are better suited for visual acuity.

However, the sexual dimorphism we observed in the wings of *J. evagoras* appears to extend to the arrangement of their ommatidia as well. Although all patches of all studied individuals had at least 20% of their ommatidia suitable for PD (Fig. 7E), female eyes had more ommatidia capable of PD than did male eyes at all elevations, although differences in PD were less pronounced at higher elevations in the eye (Fig. 7E). This may be related to the fact that while females are relatively short lived, often mating, laying eggs on nearby host plants and dying in less than a week, males are longer lived, frequently flying long distances in search of widely dispersed host plants with female pupae (Elgar and Pierce, 1988). In addition, while females typically mate only once in their lifetime, and are frequently seen rejecting the advances of males in a characteristic ‘rejection response’ (Movie 1), the high variance in male reproductive success means that although most individuals never succeed in mating, some males mate multiple times, often attempting to mate with any individual (male or female) immediately after eclosion (Elgar and Pierce, 1988; Hughes et al., 2000). Thus, if polarized patterns represent an honest signal of mate quality, choosy females may be better served by enhanced perception of polarization, whereas the more indiscriminate males may benefit from enhanced visual acuity and flight control in their wide-ranging search for new mating opportunities, using more coarse polarization vision as a means of orienting towards conspecifics at longer range. Females of *J. evagoras* may also rely upon polarization detection in the search for suitable ant symbionts and/or host plants for oviposition, as observed in females of *P. aegeus* (Kelber et al., 2001).

Whatever the exact reasons, the distribution of robust polarization detectors in the ommatidial arrays of *J. evagoras*, exploiting the heterogeneity in rhabdomeric alignment of adjacent ommatidia, shows that they are in all cases sufficiently numerous and diverse for an array of their second-order axons to support polarization vision of polarized patterns in both sexes. We here present theoretical evidence to support this hypothesis, but additional confirmation can only come from further electrophysiological and behavioral experiments.

Our discovery of misaligned ommatidia in *J. evagoras* raises questions. Do ommatidial arrays of other species of Lepidoptera also contain many misaligned ommatidia, and could this be correlated with different aspects of their life histories? Could misaligned ommatidia be found in eyes of other taxa of flying insects?

## Supplementary Material

10.1242/jexbio.244515_sup1 Supplementary information
