# Supplementary material for: A hypothesis for robust polarization vision: an example from the Australian imperial blue butterfly, Jalmenus evagoras
Source: J Exp Biol. 2023 Apr 13;226(7):jeb244515. doi: 10.1242/jeb.244515 (PMC10120070; doi:10.1242/jeb.244515)
Supplement: Supplementary information [file jexbio-226-244515-s1.pdf]

Figure S1

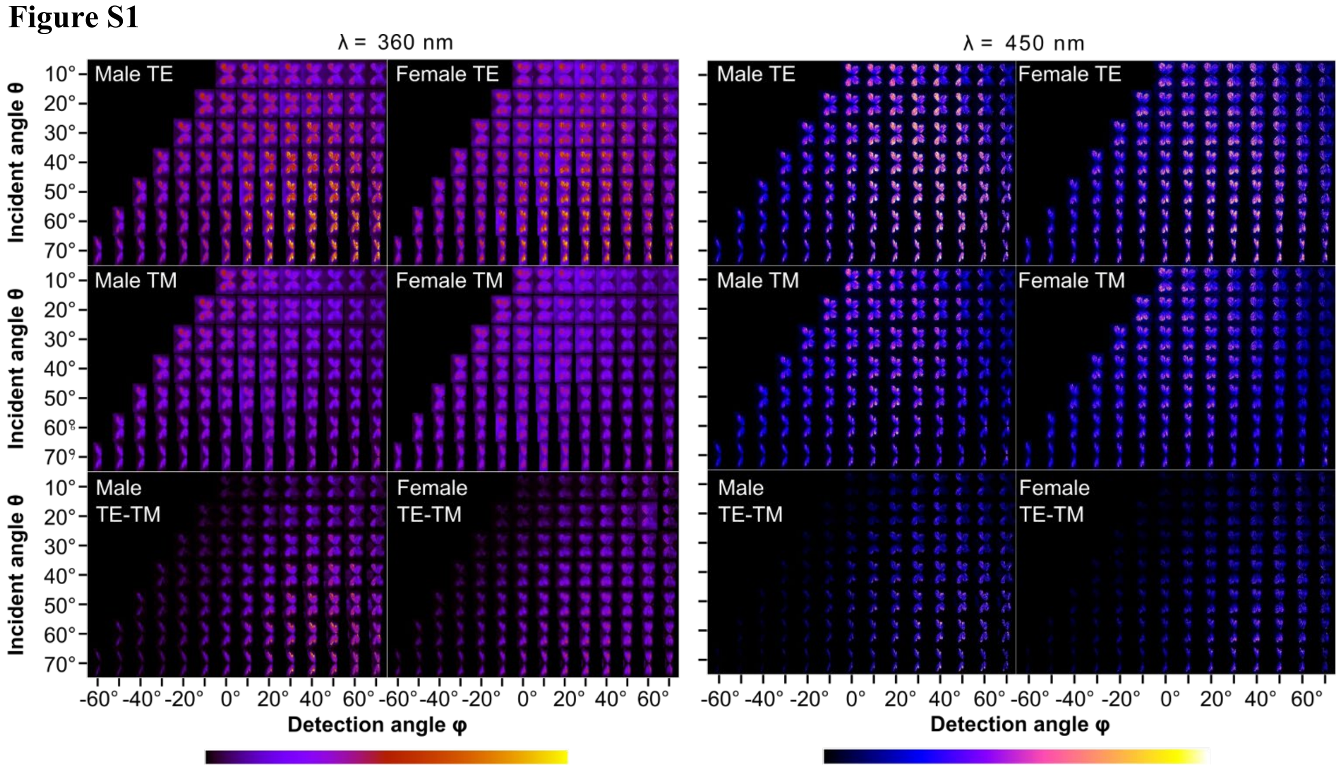

Fig. S1. Polarization images of a male and a female pinned specimen at λ=360 nm (left) and λ=450 nm (right). Intensity of TE, TM components and their difference are plotted in heatmaps. Columns (from left to right): Incident angle θ=-60°~70°. Rows (from top to bottom): Detection angle φ=10°~70°.

Journal of Experimental Biology • Supplementary information

Figure S2

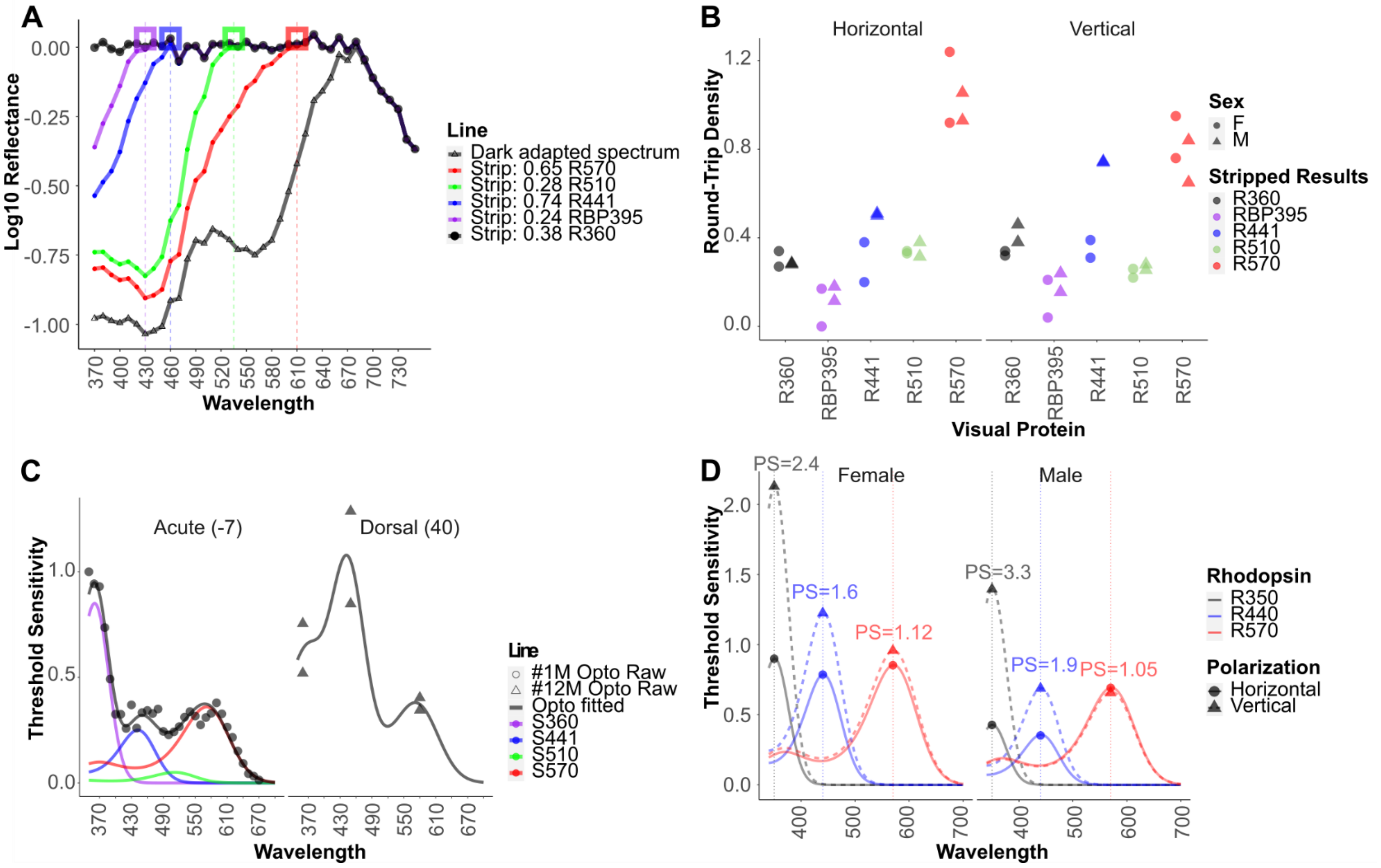

Fig. S2. Polarizational sensitivity and relative abundance of *Jalmenus evagoras* visual pigments

A) Retinal densitometry 'stripping' procedure. The average abundance of each visual pigment can be estimated computationally, given the known absorbance profiles of lepidopteran visual pigments (Bernard template, Palacios et al. (1996)) for a given peak sensitivity and the bright, spectrally neutral reflectance of the underlying tapetum (Liénard et al. 2021). Assuming that decrease in the intensity of reflected light is solely driven by the relative abundance of visual pigments present in *Jalmenus* rhabdoms, the negative contribution to log10 reflectance (absorbance) of each visual pigment can be subtracted from the measured tapetal reflectance spectrum, proceeding sequentially from longer to shorter wavelength visual pigments, until the residual reflectance is spectrally flat (i.e. an approximation of the spectrally flat averaged tapetal reflectance spectrum). The final optical densities represent the averaged abundance of the various visual pigments in the measured patch of ommatidia. Dashed vertical lines represent the wavelengths of ‘stripping targets’ for each series of stripping, which correspond to the lower limits of absorption of the nearest shorter-wavelength visual pigment. **B)** Estimated round trip optical densities (averaged abundance) of visual pigments in the medio-equatorial acute zone of two male and two female *Jalmenus evagoras* individuals using the ‘stripping’ technique described in A). **C)** Averaged threshold sensitivity of pupillary responses in two male *Jalmenus evagoras* individuals. Black lines are computational fits to the experimentally measured patch-averaged pupillary response threshold sensitivity (open circles: male #1M, acute zone; triangles: male #12M, dorsal zone). Black fitted lines are template-fitted sensitivity of each individual rhodopsin (colored lines) that together best fit the experimental data**. D)** Averaged threshold sensitivities of eye patch pupillary responses in a male and female *Jalmenus evagoras* are higher in the ultraviolet and blue than at longer wavelengths. Y-axis is the threshold sensitivity to linearly polarized light near the rhodopsin lambda-max wavelengths, as determined by the stimulation needed to elicit a criterion pupillary response of ~3% for both horizontally (circles, solid lines) and vertically (triangles, dashed lines) polarized flashes of light. Averaged patch polarizational sensitivities are denoted by the labels above each peak. Plotted curves are template functions for each rhodopsin class, scaled by their measured relative sensitivities.

Journal of Experimental Biology • Supplementary information

Figure S3

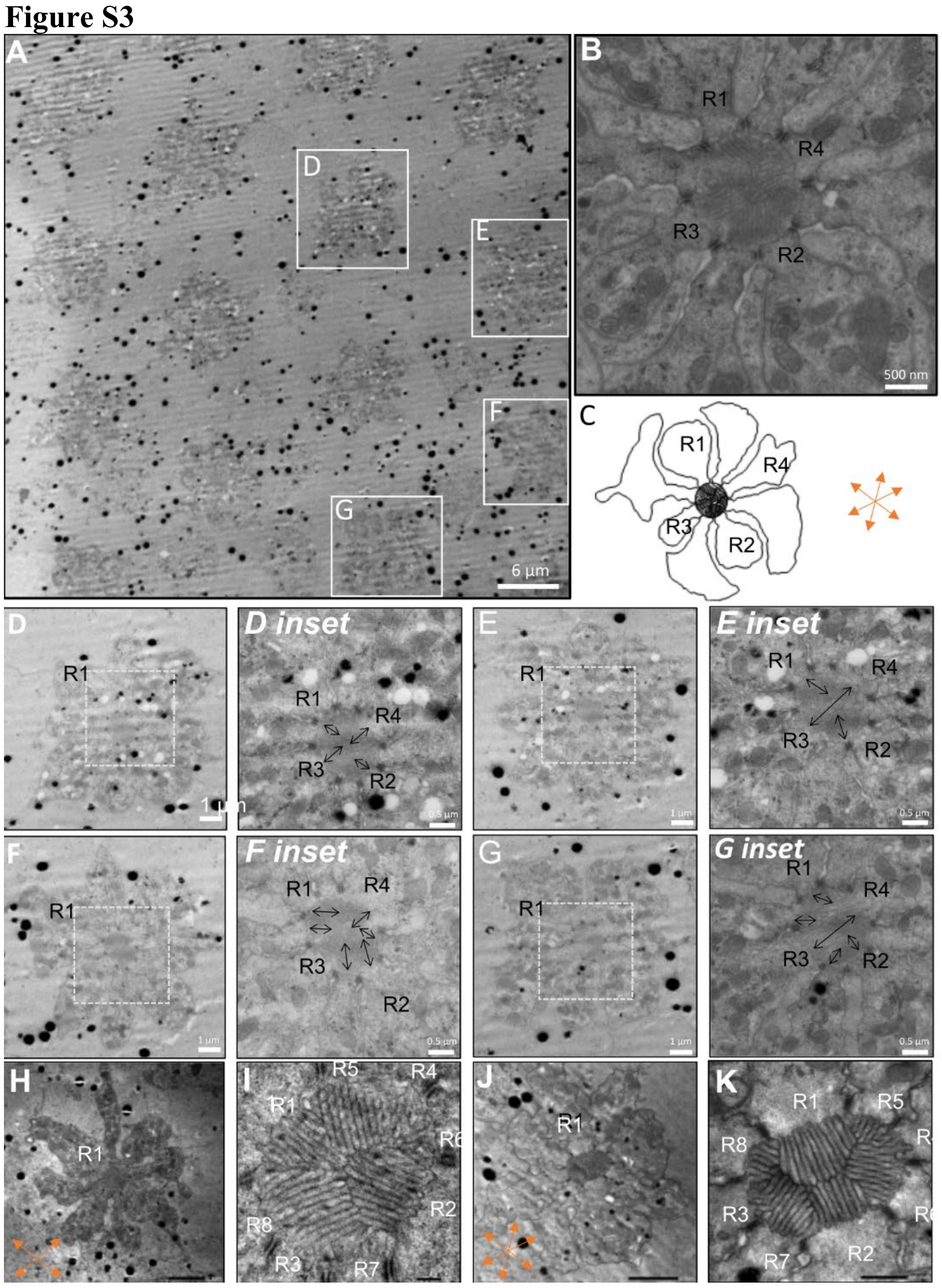

Fig. S3. Histology of *Jalmenus evagoras* ommatidial structure. A) Overview of several ommatidia in a male dorsal eye at 30 degrees elevation and at 120 microns depth from the cornea where R1-R2 cell microvilli contribute large amounts of membrane-bound blue and UV rhodopsins. All photoreceptor cells R1-R8 are visible but contribute variable numbers of microvilli, with R1-R2 being main contributors. **B)** Ommatidium in the same region as in A with multiple principal axes. **C)** Schematics of ommatidium in B) with detailed microvillar orientations. **D-G)** Individual ommatidia from overview in A) and insets with relative photoreceptor cell orientation. For illustration purposes, four adjacent ommatidia were chosen, two of which show 2 main microvillar axes (D-E), and two of which show more than two microvillar axes **(F-G)**. H-K) High resolution exemplar ommatidia showing 2 or 3 microvillar cell orientation at 150 microns from the cornea. **H)** Individual ommatidium and inset **I)** showing 2 principal microvillar axes, similarly to ommatidia D and E. **J)** Individual ommatidium and inset **K)** showing more than 2 principal microvillar axes, similar to ommatidia B, F, and G. The relative orientation of all ommatidia with respect to one another, and the eye overall, is conserved across all images to allow angular comparisons. Microvillar axes are schematized with orange arrows. In order to visualize rhabdomeric microvillar orientations in this image, brightness and contrast in this figure has been uniformly increased.

Journal of Experimental Biology • Supplementary information

Figure S4

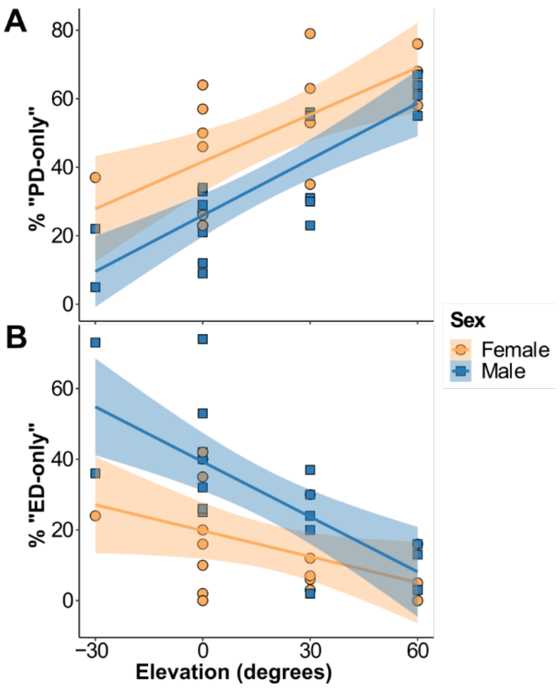

Fig. S4. Number of "ED-only" and "PD-only" ommatidia show similar patterns of distribution as more general ommatidia. A) Ommatidia that would likely be suitable for polarization-detection but not suitable for edge detection ("PD-Only") significantly increase with increasing elevation in the eye (T=26.714, p=0.0001). Overall, females exhibit a significantly larger amount of PD ommatidia than males (T=8.823, p=0.008). **B)** Ommatidia that are only suitable for edge detection significantly decline with increasing elevation in the eye (T=17.194, p=0.0001). Males show a significantly higher overall amount of such ommatidia at all elevations (T=6.761, p=0.02). For all graphs, males are plotted in blue while females are plotted in orange. The smoothed conditional means are plotted for the interaction effect of Sex and Elevation from our mixed-effects linear model analysis, with 95% confidence bands calculated using the Kenward-Rogers approximation for fixed-effect standard error.

Supplementary references

Liénard, M.A. et al., 2021. The evolution of red color vision is linked to coordinated rhodopsin tuning in lycaenid butterflies. *Proceedings of the National Academy of Sciences*, 118(6).

Palacios, A.G., Goldsmith, T.H. & Bernard, G.D., 1996. Sensitivity of cones from a cyprinid fish (Danio aequipinnatus) to ultraviolet and visible light. *Visual neuroscience*, 13(3), pp.411–421.

Journal of Experimental Biology • Supplementary information

Table S1. Model goodness-of-fit details for linear model analysis of degree of polarization by 'angular contrast.' The 'function' column describes the transformations of the response data applied to linearize the relationship between the response and predictor variables. 'Formula' describes the exact lmer model call used to implement these transformations. A series of different calculations of model R-squared include the overall model R-squared ('R2'), as well as the marginal ('Marg_R2'), Conditional ('Cond_R2'), Adjusted ('Adj_R2') and predicted R-squared ('Pred_R2') calculations, which measure different aspects of model goodness of fit. 'Df' contains the model degrees of freedom, while 'AIC' and 'BIC' columns correspond to the Akaike and Bayesian information criteria, and logLik contains the log-likelihood of the model fit, all returned from the anova function call on lme4 model objects in R (Bates et al. 2015). Final models selected for further analyses are shown in bolded rows

Data	Model	Function	Formula	R2	Marg_R2	Cond_R2	Adj_R2	Pred_R2	Df	AIC	BIC	logLik
**Blue DOP**	**model6**	**sqrt(y)=x+x^2+x^3**	**sqrt(DOP_Blue) ~ Sex * poly(Angular_contrast, 3) + (1 | Individual)**	**0.89**	**0.82**	**0.89**	**0.89**	**0.88**	**10**	**-1356.8**	**-1317.0**	**688.4**
Blue DOP	model5	sqrt(y)=x+X^2	sqrt(DOP_Blue) ~ Sex * poly(Angular_contrast, 2) + (1 | Individual)	0.86	0.79	0.86	0.86	0.85	8	-1269.6	-1237.7	642.8
Blue DOP	model3	(y)^2=x	(DOP_Blue)^2 ~ Sex * Angular_contrast + (1 | Individual)	0.72	0.63	0.71	0.71	0.70	6	-1237.3	-1213.4	624.6
Blue DOP	model2	sqrt(y)=x	sqrt(DOP_Blue) ~ Sex * Angular_contrast + (1 | Individual)	0.82	0.75	0.82	0.82	0.81	6	-1176.9	-1153.0	594.4
Blue DOP	model1	y=x	DOP_Blue ~ Sex * Angular_contrast + (1 | Individual)	0.80	0.72	0.79	0.80	0.78	6	-1066.2	-1042.3	539.1
Blue DOP	model4	log2(y)=x	log2(DOP_Blue) ~ Sex * Angular_contrast + (1 | Individual)	0.81	0.75	0.81	0.81	0.80	6	203.6	227.5	-95.8
**UV DOP**	**model5**	**sqrt(y)=x+X^2**	**sqrt(DOP_UV) ~ Sex * poly(Angular_contrast, 2) + (1 | Individual)**	**0.78**	**0.65**	**0.77**	**0.77**	**0.76**	**8**	**-1169.8**	**-1138.0**	**592.9**
UV DOP	model6	sqrt(y)=x+x^2+x^3	sqrt(DOP_UV) ~ Sex * poly(Angular_contrast, 3) + (1 | Individual)	0.78	0.65	0.77	0.77	0.76	10	-1169.3	-1129.5	594.7
UV DOP	model2	sqrt(y)=x	sqrt(DOP_UV) ~ Sex * Angular_contrast + (1 | Individual)	0.51	0.39	0.50	0.51	0.48	6	-874.3	-850.4	443.1
UV DOP	model3	(y)^2=x	(DOP_UV)^2 ~ Sex * Angular_contrast + (1 | Individual)	0.46	0.29	0.44	0.45	0.42	6	-835.5	-811.6	423.7
UV DOP	model1	y=x	DOP_UV ~ Sex * Angular_contrast + (1 | Individual)	0.50	0.36	0.49	0.50	0.47	6	-722.0	-698.1	367.0
UV DOP	model4	log2(y)=x	log2(DOP_UV) ~ Sex * Angular_contrast + (1 | Individual)	0.49	0.39	0.48	0.49	0.46	6	438.8	462.7	-213.4

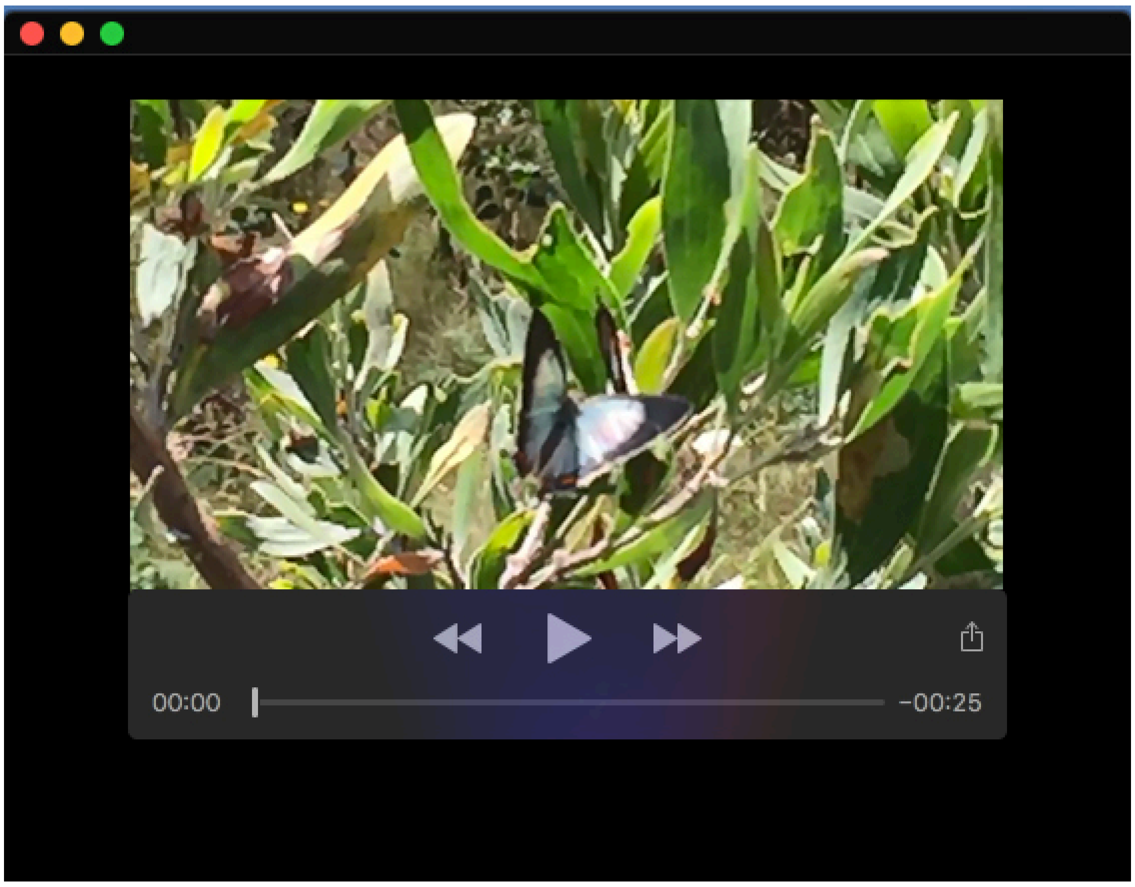

Movie 1. *Jalmenus evagoras* female exhibiting a "rejection response" towards an advancing male. Video is taken on an Apple iPhone 6 using the 'slow-motion' video capture and edited in Adobe Premiere Pro 2020 to increase sharpness and clarity using the "unsharp mask" and "convolution kernel sharpen" functions.

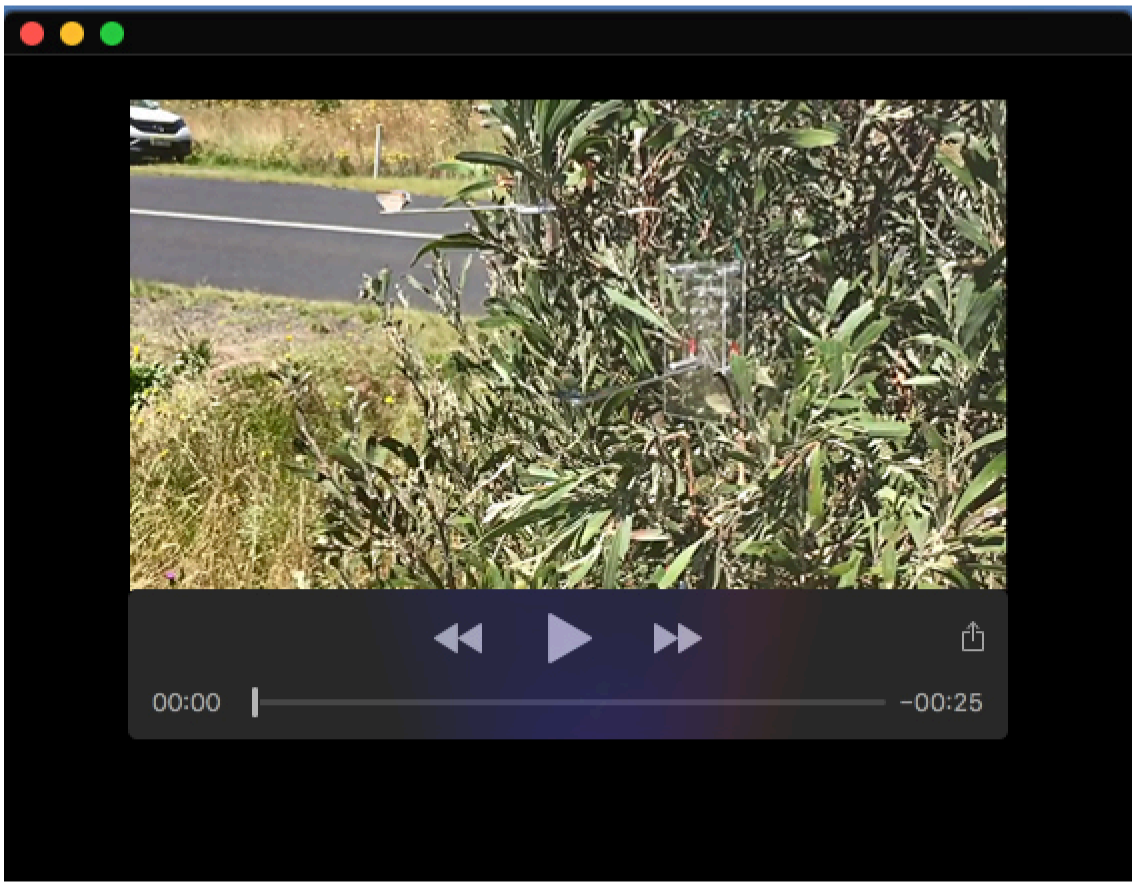

Movie 2. *Jalmenus evagoras* male exhibiting a typical inspection response towards a robotic flapper. During the polarization discrimination behavioral experiments, only responses that showed all the features exemplified in the video above were recorded: a clear deviation from the previous flight path, a pause to inspect (sometimes brief) and then resumption of normal flight. Video is taken on an Apple Iphone 6 using the 'slow-motion' video capture and edited in Adobe Premiere Pro 2020 to increase sharpness and clarity using the "unsharp mask" function.
